# Ecotoxicity of Caffeine as a Bio-Protective Component of Flax-Fiber-Reinforced Epoxy-Composite Building Material

**DOI:** 10.3390/polym15193901

**Published:** 2023-09-27

**Authors:** Klára Kobetičová, Jana Nábělková, Viktor Brejcha, Martin Böhm, Miloš Jerman, Jiří Brich, Robert Černý

**Affiliations:** 1 Department of Materials Engineering and Chemistry, Faculty of Civil Engineering, Czech Technical University in Prague, 166 29 Prague, Czech Republic; viktor.brejcha@fsv.cvut.cz (V.B.); martin.bohm@fsv.cvut.cz (M.B.); milos.jerman@fsv.cvut.cz (M.J.); jiri.brich@fsv.cvut.cz (J.B.); cernyr@fsv.cvut.cz (R.Č.); 2 Department of Urban Water Management, Faculty of Civil Engineering, Czech Technical University in Prague, 166 29 Prague, Czech Republic; nabelkova@cvut.cz

**Keywords:** composite materials, caffeine, flax, ecotoxicity, algae, yeasts, daphnids, epoxy resin

## Abstract

Caffeine is a verified bio-protective substance in the fight against the biodegradation of cellulose materials, but its ecotoxicity in this context has not yet been studied. For this reason, the ecotoxicity of flax-fiber-reinforced epoxy composite with or without caffeine was tested in the present study. Prepared samples of the composite material were tested on freshwater green algal species (*Hematococcus pluvialis*), yeasts (*Saccharomyces cerevisae*), and crustacean species (*Daphnia magna*). Aqueous eluates were prepared from the studied material (with caffeine addition (12%) and without caffeine and pure flax fibers), which were subjected to chemical analysis for the residues of caffeine or metals. The results indicate the presence of caffeine up to 0.001 mg/L. The eluate of the studied material was fully toxic for daphnids and partially for algae and yeasts, but the presence of caffeine did not increase its toxicity statistically significantly, in all cases. The final negative biological effects were probably caused by the mix of heavy metal residues and organic substances based on epoxy resins released directly from the tested composite material.

## 1. Introduction

**Figure 1:**
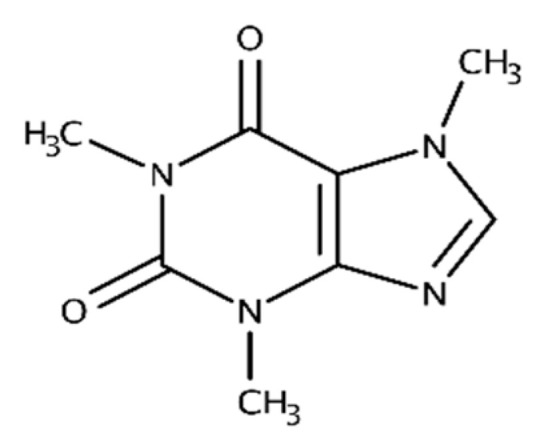
Caffeine chemical structure.

Caffeine (1,3,7-trimethyl-3,7-dihydro-1*H*-purin-2,6-dion) is a bitter, white, crystal-forming substance found in coffee beans, tea tree leaves, and kola nuts. It belongs to the methylxanthine family of bioactive substances and has been successfully used in the food, cosmetic, and pharmaceutical industries (Figure 1).

This alkaloid occurs naturally mostly in the leaves or fruits of some plants, where it has biocidal effects. Therefore, a number of scientific studies are now focusing on using its potential against various pests. Biocidal preparations that were used in the past mostly contained various metals or persistent chlorinated organic substances, the production and use of which is no longer recommended today or is directly prohibited by the international legislation on Registration, Evaluation, Assessment of Chemicals (REACH) [1] for the protection of humans and the environment and related regulations.

Its effectiveness as a biologically active substance in the fight against wood-destroying pests has been indisputably confirmed [2,3,4,5,6,7,8]. Pure caffeine or its mixtures [9,10,11,12,13] with other commercial products (siloxanes, nanoparticles, dyes, and propolis) has also been applied to wood materials under various concentrations and treatment conditions. However, its combination with other natural materials has never been tested, even though straws, bamboo, reed, or natural glues are frequently used in building or insulating materials [14,15,16,17,18,19,20].

One of the most well-known straw materials usable in civil engineering is flax. Flax fibers are relatively cheap and available in the territory of Europe. It is grown mainly in foothills areas. It is generally used for the production of food oil, textile yarn, paper production, non-sticky textiles, and sewing threads. Flax is also usable as a natural insulation material [21,22,23] or for acoustics applications [24]. If flax straw is used for construction purposes in the country of its cultivation, the ecological burdens of construction materials are reduced, which is desirable [25,26,27]. Since nowadays the production, distribution, and use of materials and chemicals that are non-toxic to humans and the environment are widely supported, it is necessary to also verify these properties for the relatively new flax-based products [28].

Composite building materials containing natural fibers are presently intensively studied because of their ecological advantages. They usually consist of a core and covering material compacted with some type of glue product. The replacement of glass fibers by natural fibers was discussed, e.g., in [29].

Ecotoxicology is a field that deals with the effects of chemical substances and various human products. If we want to evaluate the effect of a product on nature, we have to choose a suitable battery of tests with organisms in which different groups of organisms are represented [30] to obtain an answer as to whether the product is a potential threat to any of these groups. The principle of ecotoxicological evaluation consists of comparing the fitness of organisms of the so-called control group—no sample, only organisms, the culture medium, and nutrients—and in the tested sample or leachate from the sample containing organisms (the same species, the same number, age, and fitness). The degree of inhibition of the monitored parameter (increase in biomass, number of cells, survival, etc.) is then expressed as a percentage, and the potential toxicity for model groups of organisms and their species (microorganisms, plants, animals) is discussed.

The sample itself and its chemical composition, but also the properties of the environment (temperature, lighting, amount of nutrients, pH, and duration of the test) have an effect on the ecotoxicity for the monitored species (e.g., [30,31,32]).

In the case of surface materials, undesirable substances may be released into the environment due to wear and/or weather. Hypothetically, organisms living in surface waters could therefore be at risk if such materials are used in outdoor conditions. Potential pollutants can enter the water, for example, when rain is flushed into the sewage system or directly into surface waters (rivers, ponds, and lakes).

In this study, the influence of the addition of caffeine on the potential ecotoxicity of eluates from samples of flax-fiber-reinforced epoxy composites for aquatic organisms was investigated. Short-term acute toxicity tests with basic bioassays, plants (freshwater green photosynthesizing algae), decomposers of organic materials (microorganismal yeasts), and invertebrates (crustaceans), were performed. The original flax fiber material without treatment, flax fiber material treated by epoxy resin without caffeine, and flax fiber material treated by epoxy resin with caffeine (two dosages of caffeine in a mixture) were tested.

## 2. Materials and Methods

### 2.1. Materials and Chemicals

Deionized water was used as a solvent for the preparation of all aquatic solutions. Caffeine as a white, crystal powder was purchased by Sigma-Aldrich Ltd. (Prague, Czech Republic). Caffeine concentrations corresponding to 1 and 2% were prepared. These concentrations were selected on the basis of previous toxicity data with molds and fungi when they were used for the successful prevention of mold and fungus growth on selected wood species or on agar with cultured wood-rotting fungi [4,5].

**Figure 2:**
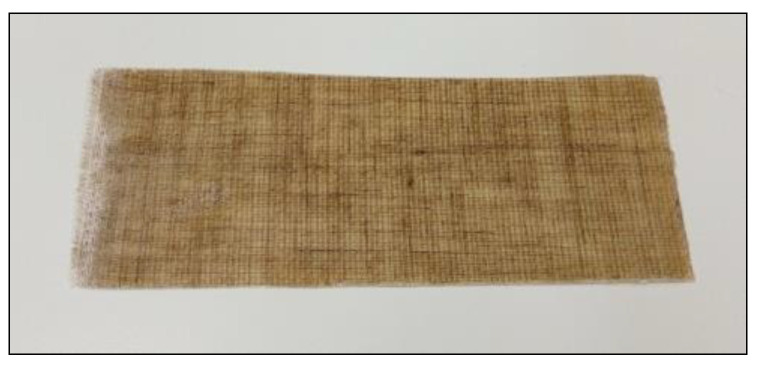
The used pure flax fibers.

Samples of bio-composite panels were made with flax fiber cloth (Figure 2), two layers of 200 g/m^2^ (Flaxdry BL200, Eco-Technilin, Valliquerville France), and epoxy resin (IB2 Epoxy Infusion Bio Resin, Easy Composites, Great Britain). The theoretical weight ratio of natural fibers and resin was set to 1:1. Infusion technology was chosen for the production process. The base surface was made of clear glass. The surface was polished and then treated by a separation system (CR-1 Easy lease Chemical Release agent, Easy Composites). After two hours it was followed by the application of two layers of flax fiber cloth in a dry form and its closure into a system of auxiliary foils and a vacuum bag. The system was settled to the vacuum level. Subsequently, it was left for 30 min in order to check the tightness of the vacuum bag. The next step was to prepare a mixture of epoxy resin at a weight ratio of 100:22. After thorough mixing, the resin was sucked into the fabric. The process of infusion of additives took approximately 8 min in total. The composite was left at a room temperature of 22 °C for the next 24 h.

**Figure 3:**
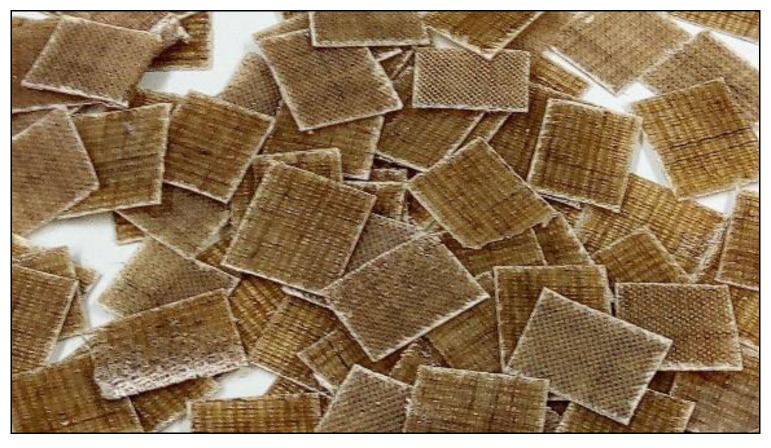
An example of a cut flax-fiber-reinforced epoxy composite with epoxide treatment and caffeine (1%).

The composite materials were then cut with a scissor into squares with an area of approximately 1 cm^2^ (see Figure 3). The squares were then used for the preparation of eluates for chemical or biological analyses.

### 2.2. Organisms

**Figure 4:**
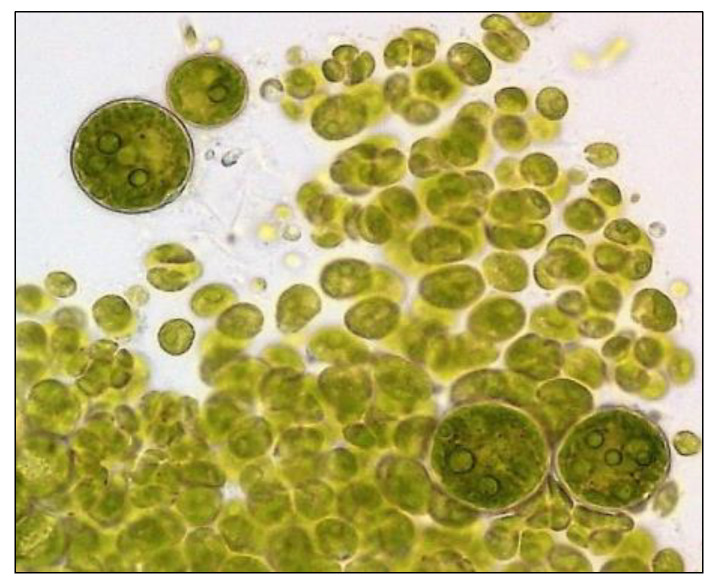
Photo of the model organism—algae (*Hematococcus pluvialis*). Videomicroscope DSM was used; magnification 600×.

**Figure 5:**
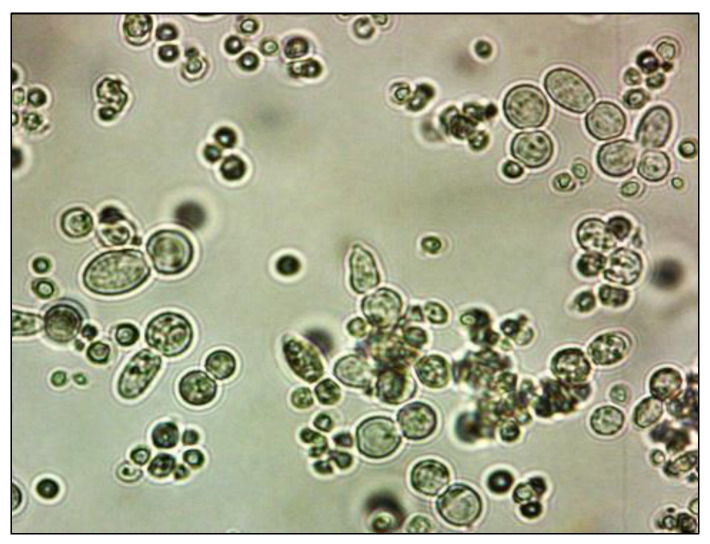
Photo of the model organism—yeasts (*Saccharomyces cerevisae*). Videomicroscope DSM was used; magnification 600×.

**Figure 6:**
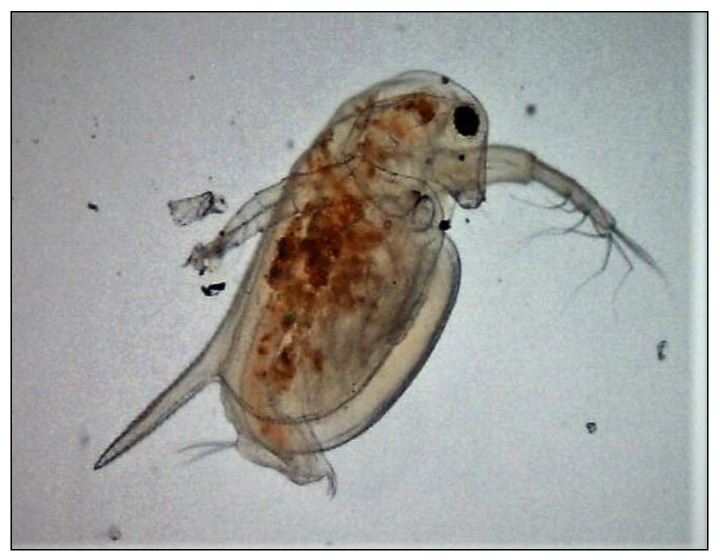
Photo of the model organism—crustacean (*Daphnia magna*). Videomicroscope DSM was used, magnification 400×.

Algae (*Hematococcus pluvialis*) were bought from CCALA (Třeboň, Czech Republic), and ephippia of daphnids (*Daphnia magna*) from MicroBio Tests, Ltd. (Ghent, Belgium). Yeasts (strain *Saccharomyces cerevisae)* were donated by the Research Institute of Brewing and Malting (Prague, Czech Republic). The photos of the three model organisms included in the present biotest battery are presented in Figure 4, Figure 5 and Figure 6.

### 2.3. Description of Eluate Preparation

Squares of each of the prepared materials were used for the preparation of eluates with deionized water at a ratio of 1:10 (material:water). The mix of material and deionized water was shaken in a REAX shaker in a vertical position for 24 h. Then, the building material cubes were passed through a sieve and the pure leachate was used for chemical and biological analyses [33].

### 2.4. Description of Caffeine Analysis

Caffeine residues in aquatic eluates were analyzed using UV–VIS spectrometry at wavelength 287 nm according to [6]. Pure deionized water (as a background), the eluate from material without the presence of caffeine, and the eluates from materials with caffeine (1 or 2%) were tested.

### 2.5. Description of Metals Analysis

Prior to metals analysis, the eluates were stabilized using nitric acid (0.1 mL of acid into 100 mL of sample) and kept refrigerated. For analysis of metals Cd, Cr, Cu, Ni, Pb, and Zn, atomic absorption spectrometry with electrothermic atomization (AAS iCE 3500Z Thermo Scientific, Waltham, MA, USA) was used. Hg was analyzed using an Advanced Mercury Analyzer (AMA 254, Altec Ltd., Prague, Czech Republic). Calibration standards were prepared by Certified Reference Materials CPAChem, Ltd., Stara Zagora, Bulgaria.

### 2.6. Bioassays

Algae: The bioassay was performed according to the appropriate OECD guideline [33]. The test was carried out in 25 mL Erlenmeyer flasks. Three replicates of 15 mL control medium and eluates were prepared. The initial algal concentration 100,000 of cells/mL was estimated by cell counting in a Bürker chamber to determine the volume of inoculum. The temperature was 22 ± 2 °C and the light/dark regime was 16/8 h (6000–8000 LUX) under stable cultivation conditions for 72 h. After exposure, the algal biomass content was determined in the Bürker chamber and the result obtained was used to calculate the growth rate.

Yeasts: A viability test with 3-[4,5-dimethylthiazol-2-yl]-2,5-difenyltetrazolium bromide was performed in plastic tubes with lids. Briefly, 5 mL of puffer (control) or sample (eluate) with yeasts *S. cerevisae* (10,000 cells/mL) were incubated at a temperature of 35 °C in the dark for 24 h. After this time period, 0.25 mL of 3-[4,5-dimethylthiazol-2-yl]-2,5-difenyltetrazolium bromide (5 g/L) was added into each of the tubes. This solution has a yellow color. The samples were incubated at a temperature of 35 °C in the dark for the next 24 h.

The next day, 5 mL of ethanol was added to each tube and the contents were mixed. The tubes with ethanol were left for the next 24 h in the dark. The principle of this test is that yeasts are able to metabolize 3-[4,5-dimethylthiazol-2-yl]-2,5-difenyltetrazolium bromide to formazan of a violet color. Only live cells in good condition are able to produce formazan. The more living cells there are in the solution, the more formazan they produce, and the more violet the solution is. The violet solutions were measured by the spectrometer at wavelength 485 nm. Two replicates were used for each sample and control. The absorbance values were overestimated on inhibition in comparison to the control—flax composite without any biocidal substance and control culture from nutrient solution [34].

Daphnia: The control medium as the eluate was aerated for 24 h before the start of the test, because a sufficient amount of oxygen is needed for the survival of daphnia (the dissolved oxygen concentration was more than 3.5 mg/L in the control and test vessels according to the OECD guideline). Then, 1-day-old daphnia from ephippia (casings, resting stages of daphnia) were placed into a control medium or the eluates to a volume of 50 mL. Ten animals were added to each of the test vessels. The animals swam in the solutions with their specific circular motion. They were not fed during the test. The measured parameter was mortality and immobilization (those animals that are not able to swim within 15 s, after gentle agitation of the test vessel, are considered to be immobilized) even if they can still move their antennae of crustaceans, which was evaluated according to the rules specified in the guideline [35]. Three replicates were used for the samples and controls. The acute test lasted 24 h.

### 2.7. Statistical Analyses

The inhibition of observed parameters for all bioassays was calculated according to Formula (1):

(1) $$I(\%)=\frac{(mc-ms)\cdot 100}{mc}$$

where I is the inhibition of the measured parameter, mc is the mean value of control, and ms is the mean value of the sample.

Growth rate was calculated according to the Formula (2):

(2) $$GR(\%)=\frac{(\ln ms-\ln m0)\cdot 100}{et}$$

where GR is the growth rate of the measured parameter, ln *m*0 is the logarithm of the mean value of the control, ln *ms* is the logarithm of the mean value of the sample, and et is the exposure time.

Dunnett’s test was performed to compare samples with controls at the α level of 0.05. The statistical analyses were performed using GraphPad Prism software (Version 3, GraphPad Software, San Diego, CA, USA).

## 3. Results

Table 1 shows the results of the chemical analysis of the leachates. Zn was below the detection limit; levels of Cd, Pb, and Hg were zero in comparison to the values for control deionized water. The main elements found in the leachates were Cr, Cu, and Ni. If we compare data for flax fibers as a background, with the data for the other eluates, the data show that material without caffeine contains more Cu and Ni than materials with caffeine. The sample with a higher caffeine concentration (2%) did not contain any metal.

The results in Table 2 indicate that residues of caffeine were detected in all samples. Caffeine was detected at the same level (0.001 mg/L) in deionized water, pure flax fibers, and flax material with epoxide and flax material with a lower concentration on the level of background. Caffeine was leached only from material treated with a higher concentration (2%). Its residues were 0.001 mg/L after the reduction in the background level of the eluate of pure flax fibers in deionized water.

**Figure 7:**
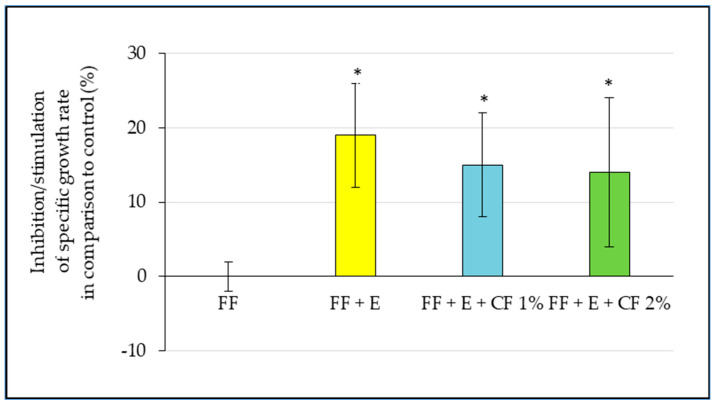
Test with algae (*H. pluvialis*)—Inhibition/stimulation of all eluates of samples in comparison with the aquatic control. The data are expressed as the inhibition of the specific growth rate of algal biomass. A total of 3 replicates were used for all samples including control. FF = Flax Fibers, FF + E = Flax Fibers treated by Epoxy resin, FF + E + C 1% = Flax Fibers treated by Epoxy resin and a Caffeine solution of 10 g/L, FF + E + C 2% = Flax Fibers treated by Epoxy resin and Caffeine solution of 20 g/L. The asterisks express the statistical significance in comparison to the control medium, Dunnett’s post hoc test at the α level of 0.05).

The results of the algal test showed a 19% toxicity of flax epoxide material and 15 or 14% toxicity of samples with caffeine. Inhibition of growth in samples with caffeine in comparison with the control medium was confirmed. The difference in toxicity was not observed between eluates from composite with 1% and 2% caffeine (Figure 7). Dunnett’s post hoc test was performed to compare samples with controls at the α level of 0.05. The compared data were not statistically significant from the control. The comparison of treated samples (blue and green boxes in Figure 7) to untreated flax composite material as a control (yellow box in Figure 7) showed no toxicity (−3 and 1%), but, again, the difference between 1% and 2% caffeine concentration was not confirmed (Dunnett’s post-hoc test at the α level of 0.05).

The absorbance values from the viability test with yeasts indicate an inhibition of 2 to 29% if we compare all samples with the control medium solution. We compare the results of the treated and untreated composite samples, the toxicity is lower (stimulation in the case of 1% addition of caffeine and only 18% inhibition in the case of the higher concentration (Table 3), similar to the algal bioassay (Figure 7A,B). For this reason, the statistical analysis for the verification of significant non-toxicity in comparison to the control sample without caffeine was not calculated.

The daphnids were not able to stay alive in the eluates from the samples, except for the eluate of pure flax fiber material without any treatment and a control medium. The mortality was thus 100% (Table 4). For this reason, the statistical analysis was not performed.

## 4. Discussion

In the present study, a new covering material composed of flax fibers, epoxide, and caffeine addition was studied from an ecotoxicological point of view. Research of this type has never been undertaken, and no one has yet tried to test the ecotoxicity of flax-based materials that should serve as surface boards for the production of sandwich-building materials. Therefore, the presented data cannot be compared to data from the literature. Nevertheless, it is possible to discuss the results in the context of chemical analyses, leachates, and established toxicity.

Although the main topic of this study is to determine whether the presence of caffeine in the material increases or decreases the toxicity of the extract, it is also necessary to ascertain the presence of other possible pollutants in the extracts. Only Cr, Cu, and Ni were found in the sample eluates (Table 1). It can be seen that the eluate from the material without the addition of caffeine contains the majority of these elements. At the same time, all the samples were leached in the same way and for the same time according to the methodology recommended for carrying out ecotoxicity tests in general. Therefore, the production of leachate should not affect the results. It is possible that the lower metal residues in the leachate were influenced by the presence of caffeine in the sample. Caffeine can probably bind these metals in the sample and prevent their leaching. Generally, caffeine interactions were confirmed for nickel [36], chromium [37], and copper [38] in the literature. Mechanisms of the interaction or binding of caffeine with parts of the composite material are unknown. They have never been described for materials of this type. For ecotoxicological assessment in the case of aquatic organisms, however, we are mainly interested in caffeine, which is able to be released from the material. It is of course possible that if we changed the length of time the material was infused, the amount of caffeine residue would be different. However, since the epoxy resin will prevent the degradation and release of substances after curing, it cannot be assumed that these are caffeine residues amounting to more than a few micrograms.

Surprisingly, the same heavy metals were found in the flax linen material. It is possible that Ni, Cu, and Cr were accumulated from the flax plants when they were growing in the field, or these elements were added intentionally or unintentionally into the flax canvas during the processing of flax or yarn. It is evident, however, that these elements were measured for the linen material itself, and if we subtract these values from the values of the others, we basically only have Ni and Cu in samples treated with caffeine left in the leaches. Even so, these are units of micrograms/L.

Nickel can be toxic in the measured levels for plants or invertebrates, but on the other hand, it is also a part of the biological molecules necessary for the lives of many organisms [36]. Copper is also an essential element, but at a higher concentration, it can also cause toxicity for aquatic and terrestrial organisms [39]. It is therefore possible that these elements caused partial toxicity of the leachates for algae and yeast or total toxicity for daphnia. Mainly their combination in the leachates could lead to some negative effects.

Caffeine was detected as a background in the deionized water that was used to make the leachates. Similar values were also found for flax fiber material, and material containing 1% caffeine. Therefore, it can be concluded that caffeine was not present in these samples. It was found only in the leachate with a higher caffeine concentration of 0.001 mg/L. Such concentrations are commonly measured in wastewater and surface waters in biomonitoring studies worldwide (e.g., [40,41,42]).

Toxicity to mussels has been demonstrated at caffeine concentrations of 0.5 ng/L to 0.5 mg/L [43]. In another study, the effect of caffeine on the survival, reproduction, and metabolic processes of daphnia (*D. magna*) was not confirmed in a laboratory ecotoxicity test until 50 ug/L [44]. The combination of some drugs with caffeine also lowered the adverse effects of drugs on *R. subcapitata* [45]. For this reason, it is possible that not only the combination of metals but also the presence of caffeine can lead to higher toxicity of eluate for yeasts (Table 3). In ecotoxicology, it is common knowledge that different species and groups of organisms tend to have different sensitivities (e.g., [46]) to the same sample and this is probably also the case with the samples in our study.

The other possible source of ecotoxicity could be the used epoxy resin or certain organic compounds such as bisphenol A or others, released from the used epoxide. Such compounds have been proven to affect organismal reproduction or metabolic disorders (e.g., [47,48,49]) but their analysis in samples is generally very complicated. Many such compounds or their combinations or degraded products can be released from samples and their identification and quantification by chemical methods is mostly impossible. In addition, the present study was focused on the addition of caffeine in the produced flax-based sample and its possible negative toxicological effects, which was another reason why some organic unknown residues were not analyzed in the studied eluates.

In general, if we studied the ecotoxicity of eluates, we had to take into account their complexity. Each of the eluates is a mixture of metals and/or organic compounds. A mixture of them leads to a final ecotoxicity to individual model species (e.g., [50]).

The striking ecotoxicity of leachates for daphnia compared to algae and yeasts has its own cause. The release of nano- or micro-particles of one’s own material, which could lead to clogging of the crustacean’s respiratory system, also comes into consideration. This effect has already been described in many studies for microplastics (e.g., [51,52,53]).

The pH values were in the range suitable for organisms to live (from 5 to 7.5) for all eluates. The acidity of control was prepared according to the appropriate media [33,34,35]. This parameter as such was not a reason for the toxicological effects.

The comparison of the ecotoxicological results (algae, yeasts, and daphnia) of the studied material evidently signals that the slight toxicity for algae and yeasts, but problematic toxicity for crustaceans, offers other possibilities for future research. It is necessary to test the leaching of the studied material (preferably without and with 1% caffeine) under conditions that will correspond to real practical use under various outdoor and indoor conditions. Mainly, it would be desirable to verify the leaching of metals and caffeine with an extended leaching time and to also study the influence of organic substances on their own toxicity, primarily in long-term experiments, focused on the reproduction and the increase in biomass with more types of organisms and species.

## 5. Conclusions

Caffeine as a potential biocidal substance was used as an additive to a newly developed flax-fiber-reinforced epoxy composite. The ecotoxicity of the composite material and its leaching into water was studied. The results indicated that caffeine was able to leach from the material if its concentration was 2%, but its residues were at a very low concentration (0.001 mg/L). The partial ecotoxicity of the original material and material with caffeine was demonstrated, but no statistically or visually significant effect on toxicity was found if a 1% caffeine concentration was used. This concentration (1%) seems to be more sufficient for biocidal purposes given its lower toxicity on yeasts. It can therefore be assumed that the analyzed composite material itself, and the organic or inorganic substances that are able to leach from it, had an observed negative effect on organisms, mainly daphnids.

## Figures and Tables

**Table 1:** The residues of metals in the eluates from flax-based building materials.

	Cd	Cr	Cu	Ni	Pb	Zn	Hg
	ug/L	ug/L	ug/L	ug/L	ug/L	ug/L	ug/L
Pure flax fibers	0.000	0.689	2.040	1.217	0.000	<0.02	0.000
Flax + EPOXIDE	0.000	0.271	5.495	3.033	0.000	<0.02	0.000
Flax + EPOXIDE + CF 1%	0.000	0.551	1.783	1.594	0.000	<0.02	0.000
Flax + EPOXIDE + CF 2%	0.000	0.000	0.000	0.535	0.000	<0.02	0.000

**Table 2:** The residues of caffeine in the eluates from flax-based building materials (mg/L). DW = deionized water, pure flax = flax linen without treatment, Flax + E = composite material with epoxide, Flax + E + CF 1% = composite material with epoxide and 1% caffeine, Flax + E + CF 2% = composite material with epoxide and 2% caffeine, E = epoxide.

	1st Replica	2nd Replica	3rd Replica	Mean	SD
Pure flax fibers	0.002	0.002	0.002	0.002	0
Flax + E	0.002	0.002	0.002	0.002	0
Flax + E + CF 1%	0.002	0.002	0.002	0.002	0
Flax + E + CF 2%	0.003	0.003	0.003	0.003	0

**Table 3:** Absorbance values with their standard deviations (SDs) of the control nutrient medium, and the eluates of the flax fibers, flax composite, and flax composite samples containing caffeine (Cf). I_1_ = Inhibition of samples in comparison to the control. I_2_ = Inhibition/stimulation of samples containing caffeine in comparison to samples without caffeine. A total of 2 replicates were prepared [35].

Sample	Control Medium	Flax Fibers	Flax Composite	Cf 1%	Cf 2%
1.	1.640	1.210	1.400	1.596	1.157
2.	1.631	1.216	1.419	1.594	1.149
Mean	1.636	1.213	1.410	1.595	1.153
SD	0.006	0.004	0.013	0.001	0.006
I_1_ (%)	-	26	14	2	29
I_2_ (%)			-	−13	18

**Table 4:** The results of the test with *D. magna*: the data are expressed as the numbers of alive individuals per a replica with 10 individuals, 3 replicates were used.

	1st Replica	2nd Replica	3rd Replica	Mean	SD	I
Control	10	10	8	9	1.15	-
Flax	10	10	9	10	0.58	-
Flax + EPOXIDE	0	0	0	0	0	100
Flax + EPOXIDE + CF 1%	0	0	0	0	0	100
Flax + EPOXIDE + CF 2%	0	0	0	0	0	100

## Data Availability

Data supporting the reported results can be found with the authors.
